# The future of general practice and family medicine in Austria

**DOI:** 10.1007/s00508-024-02422-5

**Published:** 2024-08-06

**Authors:** Susanne Rabady

**Affiliations:** Division General and Family Medicine, Department of General Health Studies, Karl Landsteiner University of Health Sciences, Dr. Karl-Dorrek-Straße 30, 3500 Krems, Austria

**Keywords:** Professional theory, Primary care physician, Generalist medicine, Defragmentation, Contextual medicine

## Abstract

General practice/family medicine has recently been recognized as a medical discipline in Austria. This paper is a short report on the prevailing understanding of its goals and subjects, comparing the Austrian perception with international definitions. It comments on shortcomings and introduces an outline for the development of a revised professional theory.

At present, there is no clear uniform image of the discipline, neither among the general public, nor among physicians, healthcare professionals or decision makers. The reason for this lies in the historical development which, with the triumph of specialization, has led to a loss of importance for generalist medicine. Now it is the fragmentation that extensive specialization entails that gives a new meaning to generalist, contextual and patient-centered medicine.

This change needs to be analyzed and understood. A description of the responsibilities, tasks and very specific methods unique to the discipline will be developed, which should enable the sensible, contemporary use of general practice/family medicine for the benefit of patients and the healthcare system.

## Introduction

### Terminology used in this text

As there are significant differences in the connotations of the terms general medicine, general practice, and family medicine between the English language definitions and the Austrian (and partly German) context, some explanations of the Austrian perception of this medical field and its terminology will be attempted in the following text. The fact that there has been no clear understanding of a medical discipline in Austria so far, is reflected in the labelling as “*Allgemeinmedizin*” (general medicine; author’s direct translation—not identical with the original English language term). Until 2015, Austrian general practitioners (GPs) received formal training only through hospital-based training with no specific training in their own field “*Allgemeinmedizin*”, not to mention any training in general practice/family medicine as understood in the international context. Additionally, most doctors of all fields completed training in “*Allgemeinmedizin*”, before specializing in their respective disciplines, so that no special training characterized and distinguished general practitioners, who were in fact addressed by the unspecific term of “*Allgemeinmediziner*” (general physician—author’s direct translation) regardless of their working context. The recent recognition as a discipline has led to a renaming in accordance with the international terminology of general practice/family medicine.

In the following, this text proposes to use, in the Austrian context, the term “*Allgemeinmedizin*” (general medicine) for the period before the recognition as the discipline of general practice/family medicine. When the term general practice or general practitioner is used for this context in this text, the physician practicing in their office is meant. In an international context the international terminology will be maintained.

### The path to an independent discipline

General practice/family medicine has only recently been recognized as a medical discipline also in Austria—as one of the last countries in Europe. Now there is an opportunity to officially define its subject, its tools and goals, and establish appropriate training based on this definition. The understanding of the discipline is generally diffuse, historically rooted in the lack of specialization: those who are “not specialized” [1], knowing something of everything—a view that has not yet completely disappeared, supplemented by the rather ambiguous attribution of being “responsible for the whole person”.

A more modern perception of GPs, also in Austria, is that they are part of primary care, serving as a first point of contact, as a coordinator who guides patients through the healthcare system [2]. This describes the administrative function of primary care, its role within the healthcare system [3], with the aim of saving costs and resources in specialized medicine. It is a public health mandate, not a description of a medical discipline and its content.

According to the European definition [4], general practice/family medicine is fundamentally different from all the specialized disciplines, and fundamentally different from all health professions. The discipline of general practice/family medicine is, in a nutshell, generalist medicine within a wide patient-related as well as community-oriented context, with the aim of defragmentation and contextualization in a highly specialized medical environment [5]. These characteristics make it well suited to fulfil the public health mandate mentioned above, but this mandate is not its first and foremost goal, as is explained later.

The source of misconceptions lies in history: in the many changes in the meaning and role of general practice, summarized here very briefly [6, 7]:

## A brief summary of the discipline’s origins and developments

The history of medicine starts at the bedside. Physicians were generalists, medicine was “general” medicine, the aim was healing individual patients in their spiritual and community context. Specialization did not exist. The ancient Greek (and later Roman) medical schools (Hippocrates and successors/pupils, Aristotle, Galen) made the first known attempts to add an early form of a scientific approach, coexisting with a holistic view, still combined with spiritualism and mysticism where knowledge was lacking.

The medieval world had lost most of the ancient knowledge, probably due to religious beliefs and restrictions. Theoretical concepts were discussed among scholars (“scholastics”), unattached to patients, separated from the practice of healing, which was dominated by magic, religion, beliefs, and prohibitions (“cloister medicine”).

Around 1000 A. D., the first Islamic Enlightenment, with Islamic and Jewish medical schools in Arab countries and Moorish Spain, brought rapid progress. This was still mostly not just holistic medicine, but a systematic search for knowledge and understanding, and the generalization of results into rules and “guidelines”, called qanoons. This period ended with the expulsion of Muslims and Jews from Spain.

Europe went back to limiting scientific efforts by religious laws and prohibitions [8]. It was the European Enlightenment from the middle of the eighteenth century, which took up Greek-Arabic knowledge that changed the world. A rapid increase in knowledge followed, leading to specialization—it could no longer be handled by a generalist.

Medicine began to focus on research, on technical means, on modern scientific methods, on curing ailments and diseases, moving away from old-fashioned healing, which was strongly based on theological and philosophical concepts.

General medicine remained, of course the generalist could not be dispensed with, but GPs remained as those who did not specialize, did not win Nobel Prizes and did not make it into the headlines with dramatic successes. Holistic medicine continued to be perceived as connected with a non-scientific approach and it was abused by esotericism, mysticism, and political extremists, at least from the 1930s onwards.

The 1950s brought a change towards a scientific understanding of general medicine. It was reborn as a medical and academic discipline in many European countries. The first academic faculties in general practice/family medicine were founded—not in Austria though, until 2001. In Austria it was the general practitioner and scientist Robert Braun, who added a milestone: a first systematic approach to general practice, concerning terminology, a description of its subject, and its typical diagnostic approach [9, 10]. Braun recognized the contextual, personal, holistic, specifically generalist nature of general practice, as well as its scientific potential and obligations. The world has changed again since Braun’s time. Cost explosion and privatization, the role of chronic diseases, multimorbidity and polypharmacotherapy, preventive and anticipatory medicine, and extended specialization are the new challenges. None of these are covered by Braun’s theories, and his terminology could not yet encompass the concept of family medicine. We now need to take his approach several steps further.

## Specialization and fragmentation as a mandate for generalist medicine

The revolutionary change in the history of medicine, and the cause for the temporary decline of generalist medicine was specialization. The medical world is still progressing to sub-specialization and sub-sub-specialization, which is exactly the cause for the re-emergence of general practice/family medicine, now scientifically based and filled with new meaning, a new definition [11]. Specialized medicine laid the foundations for the enormous progress in diagnosis and treatment, it was and is a success. A serious limitation of its usefulness lies in its fragmentation. Fragmentation leads to information deficits, overtreatment and undertreatment, conflicting treatment plans and interactions, compliance deficits and, on the part of the healthcare system, overuse of resources and cost explosion. Fragmentation of care has even been shown to lead to higher rates of potentially inappropriate medication (PIM), morbidity and mortality [12]. Patients want to be seen and recognized as individuals, as persons with their integrity and dignity, and fragmentation can cause disappointment and distrust [13].

## Conclusion: on the road to a revised professional theory

General medicine had lost core competencies to specialized medicine from the start. Now, in specialized medicine’s highly developed state, general practice/family medicine acquired new aspects, new tasks, and a new definition. Its central goals must be defragmentation, contextualization, individualization. The description of the particular specific and unique content and a concretely formulated methodology, adapted to the discipline, will be a task to be undertaken. Its objectives, from the author’s point of view, will need to be:

- To handle ambiguity (most symptoms are ambiguous as to their causes, consulting situations are frequently ambiguous as to their reasons).
- To contextualize multidimensionality (the holistic view, rationally explained as the multiple dimensions of health-related problems).
- To manage multiple illnesses (multimorbidity and comorbidity, polypharmacy, interactions between illnesses, therapies, measures, etc.).

Accordingly, the specific methodology will need a thorough as well as teachable description. This concerns:

- The diagnostic process,
- The consultation process,
- The decision-making process,
- The process of managing chronic, multiple and terminal illnesses.

Basic and indispensable tools as listed in the European definition [4] need to be included in such a framework:

- Comprehensive medical knowledge, as it has always been, supplemented by knowledge in neighboring fields, e.g. psychology, behavioral theory, sociology.
- Continuity of care: reduction of morbidity and mortality through personal continuity has been well proven [14].
- Science-oriented practice.
- Professional doctor-patient relationship.
- Person-centered care and comprehensive approach.

Family physicians apply those tools and methods intuitively, according to necessity, and developed “on the job”. Variability consequently is high. A new methodology needs to be systematically developed and described, following Brauns approach of connecting practice with scientific analysis, but taking it several steps further.

The European definition of general practice/family medicine describes characteristics, goals and core competencies. It needs to be completed by a comprehensive professional theory. Recognition as a discipline gives us the opportunity to do so, and to put the results into training and practice.
